# Mechanisms of glutamate receptors hypofunction dependent synaptic transmission impairment in the hippocampus of schizophrenia susceptibility gene *Opcml*-deficient mouse model

**DOI:** 10.1186/s13041-024-01148-9

**Published:** 2024-10-17

**Authors:** Xiaoxuan Sun, Hu Meng, Tianlan Lu, Weihua Yue, Dai Zhang, Lifang Wang, Jun Li

**Affiliations:** 1 Peking University Sixth Hospital, Peking University Institute of Mental Health, NHC Key Laboratory of Mental Health (Peking University), National Clinical Research Center for Mental Disorders (Peking University Sixth Hospital), Key Laboratory of Mental Health, Chinese Academy of Medical Sciences, Beijing, 100191 China; 2 PKU-IDG/McGovern Institute for Brain Research, Peking University, Beijing, 100871 China; 3 Changping Laboratory, Beijing, 102206 China

**Keywords:** Hippocampus, Schizophrenia, Intrinsic excitability, Glutamatergic neurotransmission, AMPA/NMDA ratio

## Abstract

Schizophrenia is a severe psychiatric disorder with high heritability, characterized by positive and negative symptoms as well as cognitive abnormalities. Dysfunction in glutamate synapse is strongly implicated in the pathophysiology of schizophrenia. However, the precise role of the perturbed glutamatergic system in contributing to the cognitive abnormalities of schizophrenia at the synaptic level remains largely unknown. Although our previous work found that Opcml promotes spine maturation and *Opcml*-deficient mice exhibit schizophrenia-related cognitive impairments, the synaptic mechanism remains unclear. By using whole-cell patch clamp recording, we found that decreased neuronal excitability and alterations in intrinsic membrane properties of CA1 PNs in *Opcml*-deficient mice. Furthermore, Opcml deficiency leads to impaired glutamatergic transmission in hippocampus, which is closely related to postsynaptic AMPA/NMDA receptors dysfunction, resulting in the disturbances of E/I balance. Additionally, we found that the aripiprazole which we used to ameliorate abnormal cognitive behaviors also rescued the impaired glutamatergic transmission in *Opcml*-deficient mice. These findings will help to understand the synaptic mechanism in schizophrenia pathogenesis, providing insights into schizophrenia therapeutics with glutamatergic disruption.

## Introduction

Schizophrenia is a devastating psychiatric disorder characterized by positive symptoms (e.g., hallucinations, delusions and disorganization), negative symptoms (e.g., social withdrawal, impaired motivation and reduced spontaneous speech) and severe cognitive impairments [1–4]. The onset of schizophrenia often occurs during late adolescence to early adulthood with an approximate prevalence of 1% in most populations worldwide [5]. The effects of schizophrenia on life expectancy and health-care systems seem to be substantial [6, 7], and understanding the molecular etiology and pathogenesis of this disorder will be helpful to develop effective and acceptable treatments to schizophrenia.

A great deal of researches has implied that dysregulation of neurotransmission and their interactions are involved in the pathophysiology of schizophrenia [8–11]. Among these, alterations in glutamatergic transmission especially the hypofunction of N-methyl-D-aspartate (NMDA) has been implicated to be fundamental to the pathophysiology of schizophrenia on account of that NMDA receptor antagonists can induce psychotic symptoms including positive, negative, and cognitive deficits in clinical, as well as the glutamatergic system is considered be an important target for therapeutic interventions [12, 13]. Disturbances of glutamatergic synaptic function might underlie abnormalities of neural circuits that involves prefrontal cortex, hippocampus and other brain areas, but the precise nature of these events are complicated and uncertain [14, 15].

The hippocampus plays a crucial role in cognitive and memory [16, 17]. Structural and functional aberrations of the hippocampus are common hallmarks of schizophrenia [18]. It’s worth noting that resting and active neuronal membrane properties of pyramidal neurons (PNs) are likely to be of functional significance within synaptic transmission and neural circuits [19]. The opioid-binding protein/cell adhesion molecule (OPCML) is a schizophrenia susceptibility gene, and *Opcml*-deficient mice appear synaptic dysfunction and schizophrenia related behaviors [20–22]. However, the role of Opcml in regulating synaptic transmission and its underlying pathophysiological mechanism in schizophrenia have not yet been fully clarified [23, 24].

To directly evaluate the means by which the connectivity state between CA3 and CA1 hippocampal pyramidal cells and their intrinsic membrane properties in *Opcml*-deficient mice, we conducted the electrophysiological approaches. Our findings offer a physiological description of that the alteration in CA1 hippocampal pyramidal cells action potentials might affect hippocampal circuits to perform specific network functions. We also uncover a synaptic mechanism of glutamatergic neurotransmission hypofunction which is mediated by alterations of postsynaptic glutamate receptors, which is involved in the pathogenesis of schizophrenia. In addition, we investigate the antipsychotic drug aripiprazole effect on glutamatergic neurotransmission in *Opcml*-deficient mice, providing potential synaptic evidence on schizophrenia therapeutics.

## Materials and methods

### Animals

All experiment procedures for the use and care of all mice were approved by and performed according to the guidelines of the Animal Care and Use Committee of Peking University (Beijing, China). The mice were housed on a 12-h light–dark cycle with food and water ad libitum. The *Opcml*^−/−^ mice were previously generated by us [25]. For electrophysiological experiments, the sequence in which animals conduct the experiments or be sacrificed is random. Meanwhile, the mice were blinded to the group allocation for the electrophysiological experiments.

### Brain slice preparation

Coronary hippocampus slices (250 μm) of mice were prepared and processed as described previously [26]. Briefly, mice were anesthetized with isoflurane and decapitated. The brain was quickly removed and sliced on a vibratome (Leica VT1200s, Wetzlar, Germany) in ice-cold cutting solution consisting of (in mM) 213 sucrose, 3 KCl, 26 NaHCO_3_, 1 NaH_2_PO_4_, 5 MgCl_2_, 0.5 CaCl_2_, and 10 glucose (adjusted to pH 7.3–7.4, 300–310 mOsm, saturated with 95% O_2_ and 5% CO_2_). The slices were incubated for 30 min at 37 °C, maintained at room temperature (24–25 °C) in a solution consisting of (in mM) 125 NaCl, 5 KCl, 26 NaHCO_3_, 2 NaH_2_PO_4_, 1.3 MgCl_2_, 2.6 CaCl_2_, and 10 glucose, bubbled with 95% O_2_/5% CO_2_ and recovered for 30 min before recording.

### Whole-cell patch-clamp recordings

Whole-cell patch-clamp recordings were performed in pyramidal neurons (PNs) from the CA1 regions by using 3–5 MΩ borosilicate glass pipettes (World Precision Instruments, 1B150F-4) pulled with a Brown-Flaming micropipette puller P-97 (Sutter Instruments Company). The recording pipettes were filled with the internal solution consisting of 145 mM KCl, 5 mM NaCl, 10 mM HEPES, 5 mM EGTA, 4 mM Mg-ATP, and 0.3 mM Na_2_-GTP (pH 7.25 with KOH, 300 to 310 mOsm) for miniature excitatory postsynaptic/inhibitory currents (mEPSCs/mIPSCs) recording, and with additional 5 QX-314 for action potential recording. The cesium (Cs) based internal solution consisting of 120 Cs-methanesulfonate, 2.8 NaCl, 5 TEA-Cl, 0.4 EGTA, 20 HEPES, 2.5 Mg-ATP, 0.25 Na-GTP (pH 7.25 with CsOH, 300 to 310 mOsm) was used for evoked EPSCs (eEPSCs) and AMPAR/NMDAR-mediated EPSCs recordings.

Under voltage-clamp mode, mEPSCs were recorded at a holding potential of − 60 mV in the presence of tetrodotoxin (TTX, 1 μM) and picrotoxin (PTX, 100 μM). Evoked EPSCs (eEPSCs) were triggered with a concentric stimulation electrode placed in the Schaffer collateral projection to CA1 with a stimulus intensity of 30 μA in the presence of PTX (100 μM). The AMPAR-mediated EPSCs were recorded in voltage-clamp mode holding at − 70 mV in the presence of PTX (100 μM) and D-2-amino-5-phosphonovalerate (D-AP5, 50 μM). Then the cell was switched to + 40 mV to evoke NMDAR-mediated EPSCs in the presence of PTX (100 μM) and cyanquixaline (CNQX, 10 μM). The NMDA/AMPA ratio was calculated by dividing the peak amplitude of the NMDA EPSC by the AMPA EPSC. Under voltage-clamp mode, mIPSCs were recorded at a holding potential of − 60 mV in the presence of TTX (1 μM), CNQX (10 μM) and D-AP5 (50 μM). The amplitude and frequency of mEPSC/mIPSC were determined for subsequent analysis. The amplitude, rise slope and decay slope of eEPSCs were calculated.

The data were digitized at 10 kHz with a 2.9 kHz low-pass filter. Recorded cells with series resistances of > 25 MΩ were rejected. All data were recorded using MultiClamp 700B amplifier and pCLAMP 10.6 software (Molecular Devices).

### Determination of passive and active electrophysiological properties

The membrane capacitance (C_m_) and membrane time constant (τ_m_) were measured after the initial break-in from giga-seal to the whole-cell configuration in voltage clamp mode. Once the C_m_ and τ_m_ were determined, the amplifier configuration was switched to current-clamp mode for action potential recording. For action potential recording, cells were held at − 70 mV to prevent spontaneous spike activity. Spikes were evoked by a series of current injection from -50 to +400 pA for 400 ms duration in 50-pA increments. The action potential kinetic properties were determined as previously reported [27]. The rheobase current required to elicit an action potential was measured. The amplitude of action potential was measured from the threshold to the peak. The after hyperpolarization (AHP) size was measured from AP threshold to the negative peak of the AHP. The AP widths were measured at halfwidth. The voltage threshold was measured in the first derivative of AP (dV/dt) considering the point where the velocity was close to 20 V/s. The interspike interval (ISI) was measured the duration between the first and the second action potential. A hyperpolarizing current pulse (400 ms) at − 50 pA was delivered to measure membrane input resistance (R_in_).

### Chemicals and drugs

All chemicals and drugs used in this study including sucrose (V900116), KCl (P3911), NaHCO_3_ (S5761), NaH_2_PO_4_ (S9638), MgCl_2_ (M9272), CaCl_2_ (C1016), glucose (G8270), NaCl (S7653), HEPES (H3375), EGTA (03777), Mg-ATP (A9187), Na_2_-GTP (51120), CNQX (C127), D-AP5 (A8054) and aripiprazole (PHR1784) were purchased from Sigma Aldrich except for PTX (C0375) was purchased from TGI and TTX (abs44200985a) was purchased from Absin.

### Drug administration

In experiments to test aripiprazole effect on synaptic transmission, aripiprazole was diluted into 0.625 mg/mL using 5% (vol/vol) Tween 80/physiological saline. We weighed the mice and then gave aripiprazole administration (*i.p.*, 2.5 mg/kg) according to the body weight while with the same volume of 5% (vol/vol) Tween 80/physiological saline as the vehicle group. Then we decapitated the mice and cut coronary hippocampus slices for later whole-cell recording after aripiprazole acute administration 30 min. The sequence of aripiprazole or vehicle administration, the brain slices preparation and patch clamp recording in wildtype and *Opcml*-deficient mice are random and blind, to avoid the random error of the brain slice quality and drug metabolism due to sequential order.

### Data analysis

Data were analyzed by Clampfit 10.6 (Molecular Devices) and Igor Pro 6.22 (WaveMetrics) software. All values are presented as mean ± SEM. Statistical comparisons were performed by GraphPad Prism 8.0.2 (GraphPad Software). Differences of *P* < 0.05 were considered significant (*, *P* < 0.05; **, *P* < 0.01; ***, *P* < 0.001).

## Results

### *Opcml*-deficient mice manifest decreased intrinsic excitability in hippocampal PNs

**Fig. 1:**
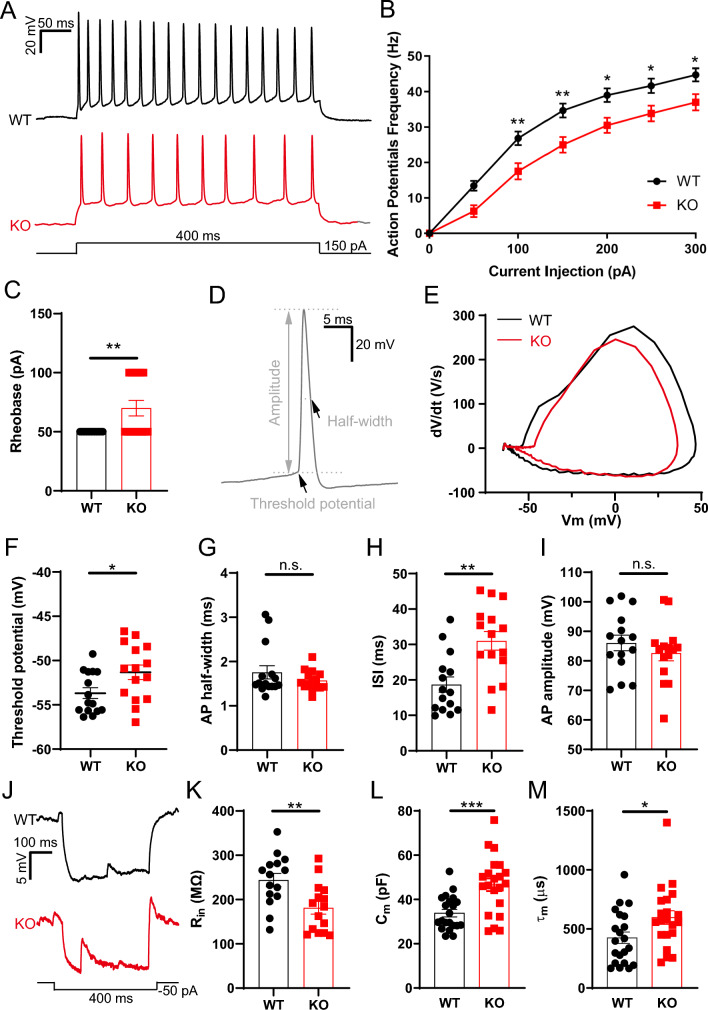
*Opcml*^−/−^ mice manifest aberrant decreased excitability of CA1 PNs. **A**, **B** Current-clamp recording of the simple spike firing of depolarized current-evoked PNs. **A** Representative traces of PCs simple spike firing evoked by a 150 pA depolarized current (duration, 400 ms) injected into the cells. **B** Summary of PN spike firing frequency evoked by a series of depolarized currents injected to the cells from 0 to 400 pA in 50 pA increments. Two-way ANOVA with Sidak’s multiple comparisons test, n = 15/3 for each group, main effect of genotype *P* < 0.0001, F (1, 196) = 51.47; main effect of injection current *P* < 0.001, F (1, 196) = 133.2; interaction effect of injection current x genotype *P* = 0.1656, F (1, 196) = 1.544. **C** Rheobase of PN spike firing evoked by a depolarized current injection in two groups. Two-tailed unpaired student’s t test, n = 15/3 for each group, t = 3.055, *P* = 0.0049. **D** Schematic illustrating parameters reflecting the intrinsic membrane properties of PNs by an evoked AP. **E** Phase plots of PNs APs evoked by a series of depolarized currents injection in *Opcml*^+/+^ and *Opcml*^−/−^ mice. **F**, **G** Summary of threshold potential and action potential half-width in two groups. Two-tailed unpaired student’s t test, n = 15/3 for each group. For (**F**), t = 2.313, *P* = 0.0283; for (**G**), t = 1.161, *P* = 0.2554. (H-I) Summary graphs showing the ISI between the first and the second AP and the AP amplitude. Two-tailed unpaired student’s t test, n = 15/3 for each group. For (**H**), t = 3.616, *P* = 0.0012; for (**I**), t = 0.9279, *P* = 0.3614. **J** Representative traces illustrating the configuration for measuring the membrane resistance (R_in_) of PNs. A hyperpolarizing current pulse (400 ms) in -50 pA was delivered to the cell to measure R_in_. **K** Summary graph showing the R_in_ of in two groups. Two-tailed unpaired student’s t test, n = 15/3 for each group, t = 3.038, *P* = 0.0051. **L** Summary graph showing the membrane capacitance (C_m_) of in two groups. Two-tailed unpaired t test with Welch’s correction, n = 21/6 for each group, t = 3.863, *P* = 0.0005. **M** Summary graph showing the membrane time constant (τ_m_) of in two groups. Two-tailed unpaired student’s t test, n = 21/6 for each group, t = 2.256, *P* = 0.0296. Data are presented as the mean ± SEM. *P* < 0.001, ***; *P* < 0.01, **; *P* < 0.05, *; *P* > 0.05, *n.s.* no significance

To examine the membrane properties which contribute to PN firing pattern and neural circuits, we first measured the membrane excitability of PNs in the CA1 by whole-cell patch-clamp recording in acute hippocampal slices (Fig. 1). A significant decrease in the simple spike firing of PNs evoked by a series of depolarized currents (from 0 to 400 pA in 50 pA increments, 400 ms duration) injected into the cells was found in the *Opcml*^−/−^ (KO) mice (Fig. 1A, B). In regard to intrinsic membrane properties, significant increases in rheobase (Fig. 1C) and threshold potential (Fig. 1F), as well as interspike interval (ISI, Fig. 1H) were observed in the PNs of *Opcml*^−/−^ mice compared with the wild-type (WT) mice. Meanwhile, the transient voltage change rate (Fig. 1E) was altered in the PNs of *Opcml*^−/−^ mice. However, no significant differences were found in the half-width (Fig. 1G) and amplitude (Fig. 1I) of AP in the PNs between the two groups. In addition to active electrophysiological properties, *Opcml*^−/−^ mice exhibited decreased membrane resistance (R_in_, Fig. 1J, K), increased membrane capacitance (C_m_, Fig. 1L) and time constant (τ_m_, Fig. 1M) compared with the wild-type mice. Altogether, these data indicate that Opcml-deficient negatively affects intrinsic excitability of hippocampal PNs in the CA1, potentially regulating the functions of hippocampal circuits.

### Excitatory synaptic transmission is impaired in hippocampus of *Opcml*-deficient mice

**Fig. 2:**
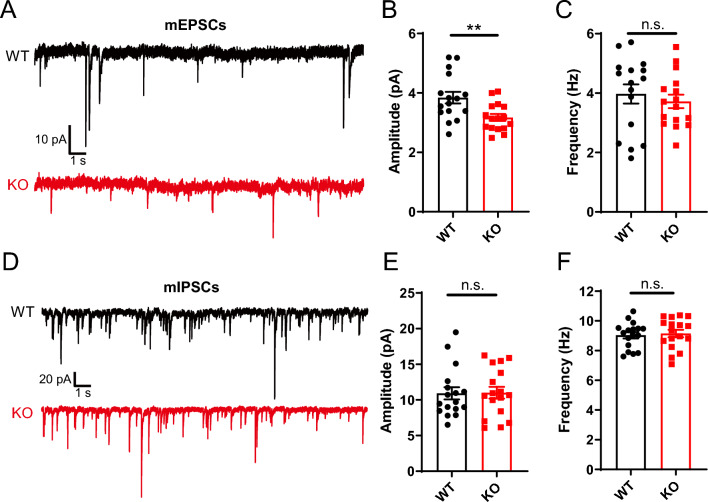
Impaired excitatory synaptic transmission reflected by miniature postsynaptic currents in *Opcml*^−/−^ mice. **A–C** Representative traces and statistics of miniature excitatory postsynaptic currents recorded from in CA1 PNs of *Opcml*^+/+^ and *Opcml*^−/−^ mice. Pooled data of mEPSCs showing KO neurons decreased in mean mEPSC amplitude but no change in mean mEPSC frequency compared to WT. Two-tailed unpaired student’s t test, n = 16/3 for each group. For (**B**), t = 2.962, *P* = 0.0059; for (**C**), t = 0.6394, *P* = 0.5274. **D–F** Representative traces and statistics of miniature inhibitory postsynaptic currents showing unchanged inhibitory synaptic transmission in KO neurons compared to WT. Two-tailed unpaired student’s t test, n = 17/3 for each group. For (**E**), t = 0.07566, *P* = 0.9402; for (**F**), t = 0.3550, *P* = 0.7249. Data are presented as the mean ± SEM. *P* < 0.01, **; *P* > 0.05, *n.s.*, no significance

To determine the synaptic transmission of hippocampal circuits, we detected the miniature excitatory postsynaptic currents (mEPSCs) recorded in CA1 PNs of *Opcml*^+/+^ and *Opcml*^−/−^ mice with tetrodotoxin (TTX, 1 μM) and picrotoxin (PTX, 100 μM) in bath. Decreased mEPSC amplitude but no change in frequency was founded in *Opcml*^−/−^ mice compared with the wild-type mice (Fig. 2A–C), indicating a reduced change in quantitative or functional of glutamate receptors in post synapse. While with tetrodotoxin (TTX, 1 μM), cyanquixaline (CNQX, 10 μM) and D-AP5 (50 μM) in bath, no significant differences were found in miniature inhibitory postsynaptic currents (mIPSCs) amplitude and frequency in *Opcml*^−/−^ mice (Fig. 2D–F), indicating an intact GABA transmission in *Opcml*^−/−^ mice.

**Fig. 3:**
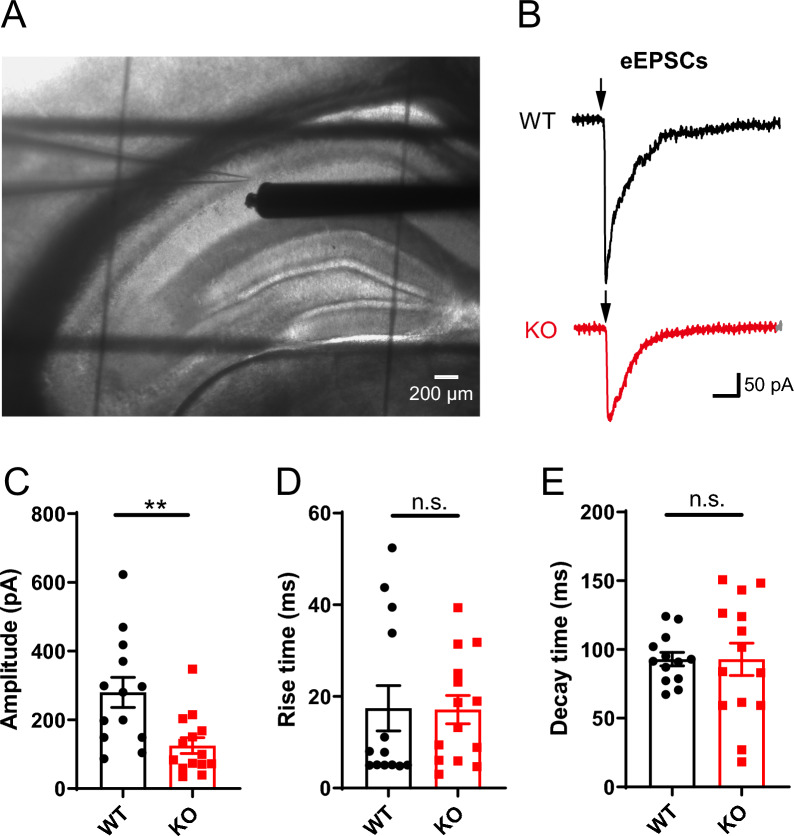
Impaired glutamatergic transmission reflected by evoked excitatory postsynaptic currents in *Opcml*^−/−^ mice. **A** Photomicrograph showing recording of E-stim (electric stimulation) evoked excitatory postsynaptic currents (eEPSCs) in hippocampal slices (bar, 200 μm). A glass electrode for recording in CA1 PN and a concentric stimulation electrode nearby Schaffer collateral were placed as illustrated. **B**–**E** Representative traces (**B**) and statistics of eEPSCs showing decreased amplitude (**C**) but unchanged rise time (**D**) and decay time (**E**) in *Opcml*-deficient PNs compared to wildtype. Two-tailed unpaired student’s t test, n = 13/5 for *Opcml*^+/+^ and n = 14/5 for *Opcml*^−/−^ mice. For (**C**), t = 3.209, *P* = 0.0036; for (**D**), t = 0.05340, *P* = 0.9578; for (**E**), t = 0.007766, *P* = 0.9939. Data are presented as the mean ± SEM. *P* < 0.01, **; *P* < 0.05, *; *P* > 0.05, n.s., no significance

Furthermore, we also performed the recording of evoked excitatory postsynaptic currents (eEPSCs) to confirm postsynaptic excitatory changes. The eEPSCs were evoked by a concentric stimulation electrode in Schaffer collateral nearby the recoding PNs in CA1 with a stimulus intensity of 30 μA (Fig. 3A, B) in the presence of PTX (100 μM). The *Opcml*-deficient PNs exhibited decreased amplitude (Fig. 3C) but unchanged rise and decay time (Fig. 3D, E) compared to wild-type, which was consistent with the reduced postsynaptic excitatory dysfunction reflected by mEPSCs.

**Fig. 4:**
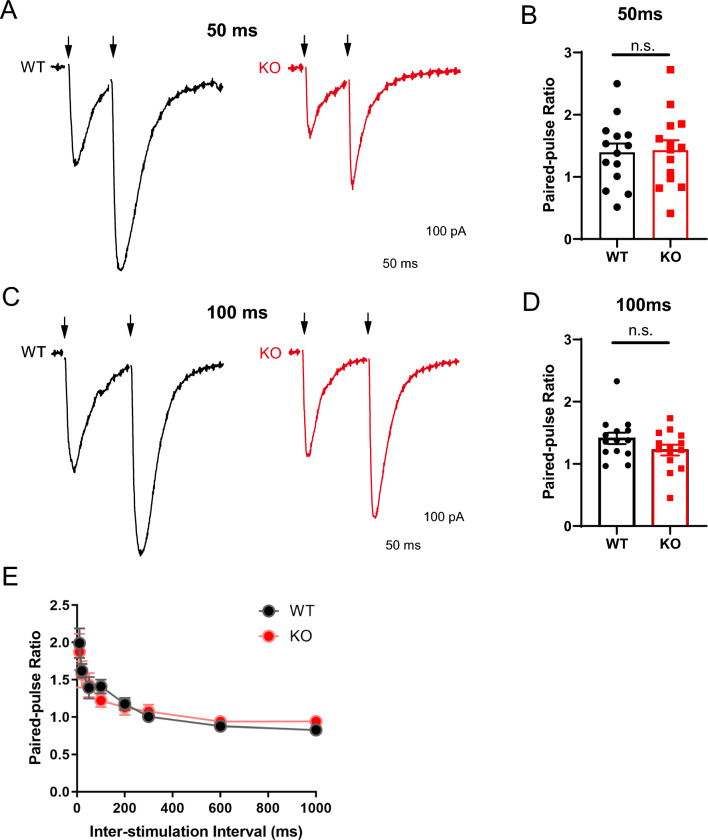
Intact presynaptic glutamate release in *Opcml*^−/−^ mice. **A**, **B** Representative EPSCs evoked by double pulses (arrowheads) with interval at 50 ms and related statistics of paired-pulse ratio. **C**, **D** Representative EPSCs evoked by double pulses with interval at 100 ms and related statistics of paired-pulse ratio. Two-tailed unpaired student’s t test for (**B** and **D**), n = 14/6 for each. For (**B**), t = 0.1648, *P* = 0.8704; for (**D**), t = 1.488, *P* = 0.1487. **E** The plot of PPR vs. different inter-stimulus intervals (10, 20, 50, 100, 200, 300, 600 and 1000 ms). Two-way ANOVA with Sidak’s multiple comparisons test, n = 14/6 for each group, main effect of genotype *P* = 0.8022, F (1, 208) = 0.06288; main effect of inter-stimulation interval *P* < 0.0001, F (7, 208) = 17.13; interaction effect of inter-stimulation interval x genotype *P* = 0.9328, F (7, 208) = 0.9328. Data are presented as the mean ± SEM. *P* > 0.05, n.s., no significance

Besides, we also recorded the EPSCs in response to paired-pulse stimulation at a variety of intervals, and used the paired-pulse ratio to evaluate the presynaptic glutamate vesicle release probability. The glutamatergic synapses in hippocampus showed robust paired-pulse facilitation (PPF) of EPSCs at representative intervals of 50 ms (Fig. 4A, B) and 100 ms (Fig. 4C, D), and the paired-pulse ratio did not alter at all recorded intervals between *Opcml*^+/+^ and *Opcml*^−/−^ mice (Fig. 4E). This was consistent with the mEPSC results, indicating that glutamatergic synapses in hippocampus of *Opcml*^−/−^ mice has unaltered presynaptic release probability.

Thus, we found a certain glutamatergic transmission defect with changes more likely to be associated with post-synapse glutamate receptors in *Opcml*-deficient mice, closely related to the functions of neural circuits in hippocampus.

### Dysfunctions of AMPA and NMDA receptors contribute to impaired glutamatergic transmission in *Opcml*-deficient mice

**Fig. 5:**
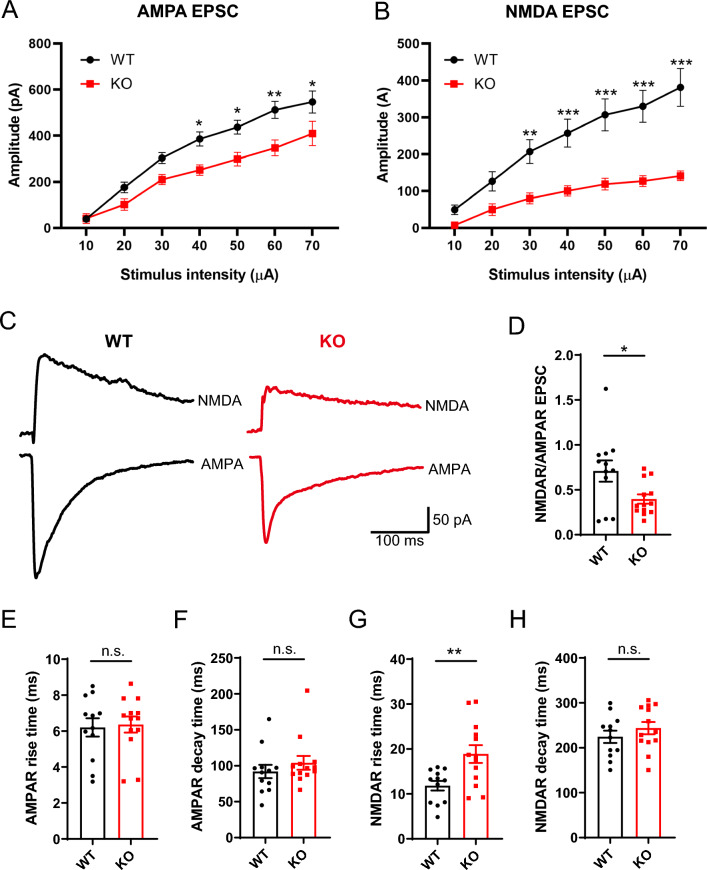
Impaired AMPA/NMDA mediated synaptic transmission function in *Opcml*^−/−^ mice. **A**, **B** Input–output curves of AMPAR and NMDA mediated EPSCs from *Opcml*^+/+^ and *Opcml*^−/−^ mice. Two-way ANOVA with Sidak’s multiple comparisons test, n = 12/5 for *Opcml*^+/+^ and n = 13/5 for *Opcml*^−/−^ mice. For (**A**), main effect of genotype *P* < 0.0001, F (1, 161) = 40.21; main effect of stimulus intensity *P* < 0.0001, F (6, 161) = 50.78; interaction effect of stimulus intensity x genotype *P* = 0.1414, F (6, 161) = 1.632. For (**B**), main effect of genotype *P* < 0.0001, F (1, 161) = 103.7; main effect of stimulus intensity *P* < 0.0001, F (6, 161) = 18.42; interaction effect of stimulus intensity x genotype *P* = 0.0035, F (6, 161) = 3.403. **C**, **D** Representative traces and statistics showing impaired AMPAR/NMDAR EPSCs and decreased NMDAR/AMPAR ratio in *Opcml*-deficient PNs compared to wildtype. Two-tailed unpaired student’s t test, n = 12/5 for *Opcml*^+/+^ and n = 13/5 for *Opcml*^−/−^ mice, t = 2.470, *P* = 0.0214. **E, F** Statistics of AMPAR mediated EPSCs rise time (**E**) and decay time (**F**) kinetics measurements of PNs in two groups. Two-tailed unpaired student’s t test, n = 12/5 for *Opcml*^+/+^ and n = 13/5 for *Opcml*^−/−^ mice. For (**E**), t = 0.2387, *P* = 0.8134; for (**F**), t = 0.9054, *P* = 0.3746. **G**, **H** Statistics of AMPAR mediated EPSCs rise time (**G**) and decay time (**H**) kinetics measurements of PNs in two groups. Two-tailed unpaired student’s t test, n = 12/5 for *Opcml*^+/+^ and n = 13/5 for *Opcml*^−/−^ mice. For (**G**), t = 3.060, *P* = 0.0056; for (**H**), t = 1.006, *P* = 0.3247. Data are presented as the mean ± SEM.* P* < 0.001, ***; *P* < 0.01, **; *P* < 0.05, *; *P* > 0.05, *n.s.* no significance

We next investigated the mechanism underlying the decreased excitatory synaptic transmission function in the *Opcml*-deficient mice. Alpha-amino-3-hydroxy-5-methyl-4-isoxazolepropionate (AMPA) and N-methyl-D-aspartate (NMDA) receptors are the main ionotropic glutamate receptors predominantly postsynaptic and are widely distributed in hippocampus. To further unravel the post-synapse glutamate receptors changes, we conducted analysis of excitatory postsynaptic currents mediated by AMPA and NMDA receptors respectively. The AMPA receptor mediated EPSCs were recorded at − 70 mV with PTX (100 μM) and D-AP5 (50 μM) in bath, while the NMDA receptor mediated EPSCs were recorded at + 40 mV with PTX (100 μM) and CNQX (10 μM) in bath. We first investigated synaptic strength by measuring input/output curves of evoked AMPA and NMDA receptor mediated synaptic responses, and both reduction of AMPA and NMDA mediated synaptic currents were detected in PNs of *Opcml*^−/−^ mice (Fig. 5A, B), suggesting cumulative reduction of AMPA and NMDAR mediated synaptic transmission in PNs of *Opcml*-deficient mice. Then we choose the stimulation strength which induced ~ 40% of largest EPSC amplitude to analysis of the NMDA/ AMPA ratio, the result showed that the ratio of PNs in the CA1 region of hippocampus was obviously declined in *Opcml*^−/−^ mice compared with the wild-type mice (Fig. 5C, D), indicating NMDA receptor mediated synaptic function impaired more heavily. We further characterized the properties of AMPA and NMDA receptors in the hippocampus of the *Opcml*-deficient mice. The rise and decay time constants of AMPA-receptor-mediated responses at − 70 mV of PNs were unaltered (Fig. 5E, F), while NMDA-receptor-mediated responses at + 40 mV of PNs exhibited an increased rise but unchanged decay time (Fig. 5G, H) in *Opcml*-deficient mice. Collectively, these results indicating that the underlying mechanism of AMPA and NMDA receptors contributing to the impaired excitatory synaptic transmission may be not entirely same.

### Administration of aripiprazole improves the impaired excitatory synaptic transmission in *Opcml*-deficient mice

**Fig. 6:**
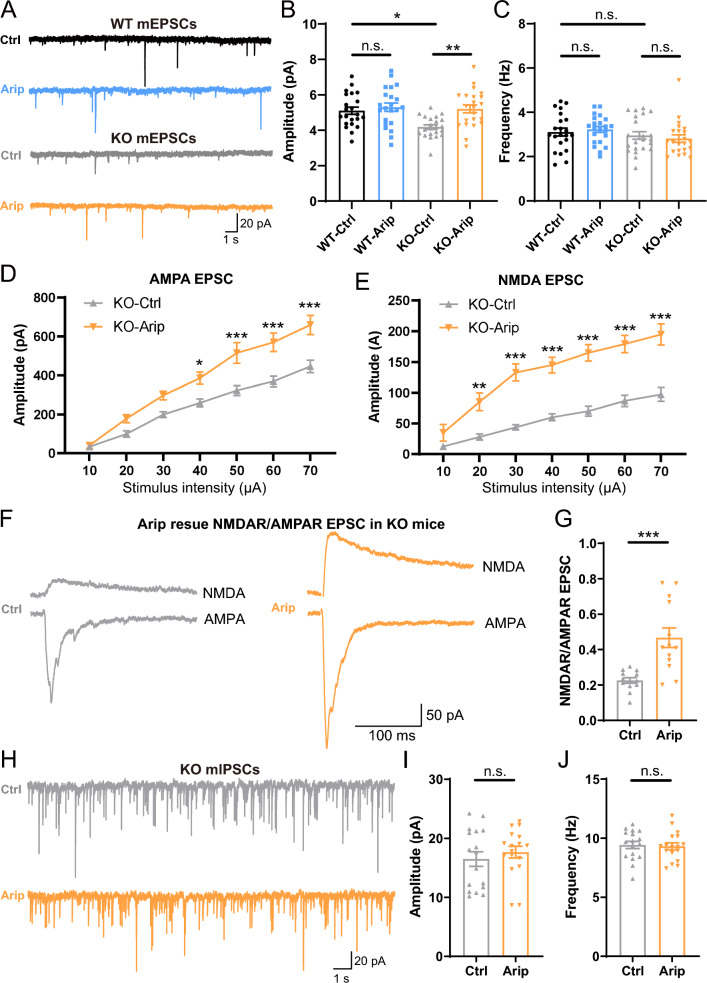
Aripiprazole rescued impaired excitatory synaptic transmission in *Opcml*^−/−^ mice. **A** Representative traces of miniature excitatory postsynaptic currents recorded from in CA1 PNs of *Opcml*^+/+^ and *Opcml*^−/−^ mice with aripiprazole (2.5 mg/kg, *i.p.*) treatment. **B**, **C** Pooled data of mEPSCs amplitude and frequency, showing aripiprazole rescued decreased mEPSC amplitude in *Opcml*-deficient PNs compared to wildtype. Two-way ANOVA with Sidak’s multiple comparisons test, n = 22/4 for each group. For (**A**), main effect of genotype *P* = 0.0143, F (1, 84) = 6.259; main effect of drug *P* = 0.0036, F (1, 84) = 8.996; interaction effect of drug x genotype *P* = 0.0430, F (1, 84) = 4.220. For (**B**), main effect of genotype *P* = 0.0865, F (1, 84) = 3.008; main effect of drug *P* = 0.9557, F (1, 84) = 0.003099; interaction effect of drug x genotype *P* = 0.4354, F (1, 84) = 0.6143. **D**, **E** Input–output curves of AMPAR and NMDA mediated EPSCs from *Opcml*^−/−^ mice with aripiprazole treatment. Two-way ANOVA with Sidak’s multiple comparisons test, n = 13/4 for each group. For (**D**), main effect of drug *P* < 0.0001, F (1, 168) = 64.77; main effect of stimulus intensity *P* < 0.0001, F (6, 168) = 73.16; interaction effect of stimulus intensity x drug *P* = 0.0064, F (6, 168) = 3.118. For (**E**), main effect of drug *P* < 0.0001, F (1, 168) = 161.8; main effect of stimulus intensity *P* < 0.0001, F (6, 168) = 29.28; interaction effect of stimulus intensity x drug *P* = 0.0087, F (6, 168) = 2.976. **F**, **G** Representative traces and statistics showing impaired AMPAR/NMDAR EPSCs and decreased NMDAR/AMPAR ratio in *Opcml*-deficient PNs were increased after aripiprazole treatment. Two-tailed unpaired t test with Welch’s correction, n = 13/4 for each group, t = 4.197, *P* = 0.0009. **H**–**J** Representative traces and statistics of miniature inhibitory postsynaptic currents in CA1 PNs of *Opcml*^−/−^ mice with aripiprazole treatment. Two-tailed unpaired student’s t test, n = 13/4 for each group. For (**I**), t = 0.7366, *P* = 0.4667; for (**J**), t = 0.2597, *P* = 0.7968. Data are presented as the mean ± SEM. *P* < 0.001, ***; *P* < 0.01, **; *P* < 0.05, *; *P* > 0.05, *n.s.*, no significance

Aripiprazole is a well-used medication in the treatment of schizophrenia because of its good therapeutic efficacy for cognitive disturbances [28]. In our previous study, we found that there was a strong association between polymorphisms in *OPCML* and aripiprazole [29]. Administration of aripiprazole could ameliorate the impaired spine maturation and schizophrenia-related cognitive behaviors in *Opcml*^−/−^ mice [22]. Here we further evaluated the effects of aripiprazole on excitatory synaptic transmission after acute administration. We performed whole-cell recording on PNs in hippocampus CA1 after aripiprazole administration (2.5 mg/kg, *i.p.*) in two groups with random order. The results showed that the decreased mean mEPSC amplitude of *Opcml*^−/−^ mice was rescued after aripiprazole administration (Fig. 6A, B), while aripiprazole had no effect on mean mEPSC frequency in both *Opcml*^+/+^ and *Opcml*^−/−^ mice (Fig. 6C). Then we measured evoked AMPA and NMDA receptor mediated EPSCs with different stimulus intensities, we found the impaired AMPAR and NMDAR mediated EPSCs (Fig. 6D–F) as well as the NMDA/AMPA ratio (Fig. 6G) in PNs of *Opcml*^−/−^ mice were all significantly increased after aripiprazole treatment. However, aripiprazole had no effect on both amplitude and frequency of mIPSCs (Fig. 6H–J) in PNs of *Opcml*^−/−^ mice. These findings suggest that aripiprazole likely to be correlative to the regulation of postsynaptic glutamate receptors’ function, providing a potential synaptic mechanism in aripiprazole therapy on cognitive disfunctions in schizophrenia.

## Discussion

As a follow-up to our previous work and taking advantage of the *Opcml*-knockout mouse model that we generated, we determined that the complete absence of Opcml leads to a less excitable synaptic network at synapses in hippocampus.

In this study, a decreased neuronal activity of CA1 PNs and alterations in intrinsic membrane properties were found in *Opcml*^−/−^ mice, and these changes may have a relationship with the substantial decrease of mature dendritic spines due to the absence of Opcml. Reduced spine densities and increased immature spines in hippocampus and other brain regions is a hallmark of schizophrenia pathology which have been previously found in patients with schizophrenia [30, 31], while spine morphological abnormity strongly corresponds to disturbance in neuronal firing [32, 33]. The spine morphology especially the length and diameter of the spine neck influences the degree and dynamics of postsynaptic Ca^2+^ elevation mediated by ionotropic glutamate receptors activation [34]. GluA2-lacking AMPA receptors and NMDA receptors are typically Ca^2+^ permeable, and their activation induces a calcium influx, which in turn could affect neuronal excitability [35, 36]. In our present work, we also found postsynaptic AMPAR/NMDAR dysfunction in CA1 PNs of *Opcml*^−/−^ mice. In addition, the spines of hippocampal CA1 neurons in *Opcml*^−/−^ mice showed a smaller head and longer neck than those of the WT mice [22], leading to difficulty in Ca^2+^ entry, collaboratively contributing to CA1 PNs hypoexcitability.

The observed decreased mEPSC or eEPSC amplitude is considered to a postsynaptic glutamatergic transmission defect, while the unchanged mEPSC frequency and robust paired-pulse ratio suggests an intact presynaptic glutamatergic transmission. The released glutamate acted predominantly upon AMPA and NMDA receptors at postsynaptic site to mediate fast excitatory transmission. Gating of the postsynaptic receptors is an interrelated factor to the time course of the postsynaptic conductance change, the number or the subunit composition change both can alter the conductance [37, 38]. The unchanged rise and decay time constants of AMPA receptor-mediated responses of PNs indicate reduced number of the AMPA receptors but no change in subunit composition. Unlike AMPA receptors, NMDA receptors displays increased rise time, this implies that maybe due to the immature state of PNs and spines [39]. More importantly, the NMDA/AMPA ratio decreased, suggesting NMDA receptors dysfunction more heavily, and this may due to a secondary effect of AMPA receptors hypoactivity [12]. AMPA and NMDA receptors play critical roles in synaptic plasticity, including long-term potentiation (LTP) and long-term depression (LTD) [40]. The dysfunction of these two glutamatergic receptors may lead to synaptic plasticity disruption, contributing to the observed cognitive impairments in *Opcml*-deficient mice.

Synapses are the fundamental information-processing units in the neuronal network functions of the brain. The formation and maturation of different synapses are important in establishing neural circuits. Microcircuits composed of excitatory pyramidal neurons and local inhibitory interneurons provide crucial roles on information processing in hippocampus [41–43]. Synapse development impairments could lead to the imbalance of excitatory and inhibitory (E/I) synaptic activity which contributes to the pathologies of several neurologic disorders. E/I imbalance in the brain is recently suggested as pathophysiological feature of underlying disorders like schizophrenia and autism, resulting in associated behavioral alterations including deficits of recognition memory [44, 45]. Opcml deficiency results in a significant decrease in glutamatergic synapse transmission but has no effect on GABAergic synapses in hippocampus, leading to a disturbance of the excitation/inhibition (E/I) balance. The E/I ratio is decreased in CA1 PNs of *Opcml*^−/−^ mice due to attenuated excitation of postsynaptic AMPAR/NMDAR dysfunction, leading to neural circuit disruption in hippocampus with consequent cognitive deficits and PPI impairments of *Opcml*-deficient mice.

Aripiprazole, considered of partial dopamine D_2_ and serotonin 5-HT_1A_ receptors agonist as well as partial serotonin 5-HT_2A_ receptors antagonist, is approved for schizophrenia treatment in clinical, but the underlying mechanism is not well understood yet [46, 47]. Here we found the aripiprazole can rescue the impaired excitatory synaptic transmission reflected by mEPSCs and evoked AMPAR/NMDAR mediated EPSCs in *Opcml*^−/−^ mice while has no effect on inhibitory synaptic transmission, implying aripiprazole may have an interaction with glutamate receptors somehow. Previous studies have reported that glutamate receptors have interactions with other receptors, including 5-HT_1A_, 5-HT_2A_ and D2 receptors. Activating 5‐HT_2A_ receptor can enhance NMDA receptor‐mediated glutamate currents [48] while activating 5‐HT_1A_ receptor suppress NMDA receptor‐mediated glutamate currents [49]. D2 receptor activation inhibits the phosphorylation of GLUA1‑containing AMPA receptor and its membrane expression through PI3K pathway [50, 51]. But these findings run counter to our results in rescue of glutamate receptors hypofunction by aripiprazole. However, there’s also a report showing that 5-HT_1A_ and NMDA receptors together with CaMKII formed a synergistic triad in clozapine functions on neuronal activity, supporting that activating 5-HT_1A_ will augment NMDA receptor function in post-synapse [52]. For now, how aripiprazole regulate glutamate transmission still remains unclear, and this will be investigated in further study.

**Fig. 7:**
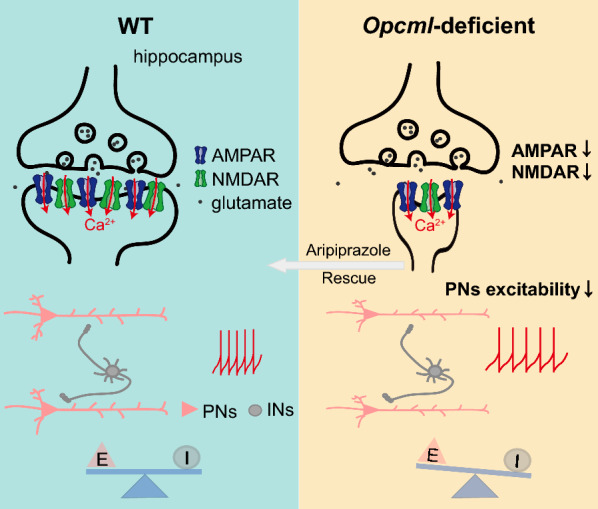
Synaptic mechanism cartoon of glutamatergic transmission impairment in the hippocampus of a schizophrenia mouse model. Dysfunctions of AMPA and NMDA receptors leads to decreased neuron excitability and impaired excitatory synaptic transmission in hippocampus of *Opcml*-deficient mice, which can be rescued by aripiprazole administration

Taken together, we have provided physiological evidences that hippocampal hypofunction of glutamate receptors negatively regulates excitatory PNs activity and glutamate transmission in *Opcml*-deficient mice (Fig. 7). These results partially explain the spine morphological abnormity and cognitive deficits which we found in our previous work of this schizophrenia mouse model. The decrease of mature spines due to Opcml deficiency results to PNs hypoexcitability and postsynaptic AMPAR/NMDAR dysfunction, leading to glutamatergic transmission defect and E/I imbalance of hippocampal neural circuits, which may further contribute to the schizophrenia related behavioral disorders in this animal model. Aripiprazole can improve the decreased EPSCs and further E/I imbalance due to impaired glutamatergic transmission in *Opcml*-deficient mice. The synaptic mechanism will help to uncover the secrets of schizophrenia pathogenesis, also providing potential targets in schizophrenia therapeutics with glutamate transmission hypofunction.

## Data Availability

The data and materials that support the findings of this study are available from the corresponding author upon reasonable request.
